# Midline vs. flank laparotomy- criteria for choosing the optimal surgical technique for uterine torsion correction in the mare

**DOI:** 10.1186/s12917-025-04883-w

**Published:** 2025-09-24

**Authors:** Jan Samsel, Ozan Gündemir, Tomasz Szara, Maciej Witkowski

**Affiliations:** 1 Equine Clinic in Warsaw Racetrack, Warsaw, 02-684 Poland; 2 Department of Anatomy, Faculty of Veterinary Medicine, Istanbul University-Cerrahpasa, Istanbul, 34500 Turkey; 3 Department of Morphological Sciences, Institute of Veterinary Medicine, Warsaw University of Life Sciences— SGGW, Warsaw, 02-776 Poland; 4 Faculty of Veterinary Medicine, Department of Diagnostics and Clinical Sciences, Krakow, 30-248 Poland

**Keywords:** Mare, Uterine torsion, Midline laparotomy, Flank laparotomy

## Abstract

Uterine torsion in mares belongs to maternal pregnancy disorders, accounting for 5–10% of complications in the last trimester of pregnancy. Two surgical techniques for repositioning uterine torsion are used: flank laparotomy in local anesthesia on a standing mare (SFL) and midline laparotomy carried out under general anesthesia (MI). The study aims to present the exact protocol used by the authors to qualify a mare with uterine torsion for surgery using one of the above-mentioned methods.

A total of 19 mares were operated on, of which 13 underwent midline laparotomy under general anesthesia, and the flank approach in a standing position operated on 7. Of the seven mares operated on in standing position under local anesthesia, six recovered and gave birth to healthy foals. In one of the operated mares by this approach, repositioning of the uterus was unsuccessful, and torsion was finally resolved after performing a laparotomy in the midline. Out of 13 operated mares in the midline (including the last-mentioned case), seven mares recovered and gave birth to normal foals. Another mare underwent c.s. because of the impossibility of twisted uterus reposition, but its outcome was good. 2 other mares with dead fetuses at admission underwent c.s. as well. One of them was in a critical general condition and died during surgery; the outcome of the other one was good. 3 following mares from this group aborted dead fetuses during the first week after the operation. One of them was euthanized after abortion, because of post-operative complications, the other two recovered without complications.

Based on their own experience and available literature, the authors currently use the following key when selecting an appropriate surgical technique for uterine torsion repositioning in the mare:

A mare of a balanced character promising approval of the procedure in sedation and local anesthesia with pregnancy up to 320 days with a living fetus and no apparent advanced circulatory changes within the uterine wall and/or broad ligament (diagnosed by rectal palpation and/or ultrasound examination) and no suspicion of comorbidities – flank approach in standing position (standing flank laparotomy SFL).

Nervous, unpredictable mare, pregnancy over 320 days, dead fetus and/or severely compromised uterine wall, suspicion of concomitant abdominal problems – midline incision in general anesthesia (MI).

## Introduction

Uterine torsion in mares belongs to maternal pregnancy disorders, accounting for 5–10% of complications in the last trimester of pregnancy [1, 2]. Most often, cases of uterine torsion occur in mares from the 8th month of pregnancy, although the described torsions arise as early as the 110th- 130th day of pregnancy [3, 4]. The main clinical sign of uterine torsion is the occurrence of pain of varying severity in the abdominal area. These signs are accompanied by anxiety, lack of appetite, excessive sweating, and frequent urination. Increased mobility of the fetus in the flank area of mares and premature lactation can be observed [5]. The most serious complications of uterine torsion are rupture of the uterine wall, placental abruption, fetal death, endotoxic shock, peritonitis, and death [6, 7]. Differential diagnosis should include gastrointestinal colic and signs of impending parturition or abortion. In some cases, colic signs may be poorly expressed, transient after analgesics, while in others, pain can be extreme. Reported cases indicate the condition may persist for weeks [8]. However, with advanced torsions, the condition progresses acutely, rapidly endangering the fetus and mother. Unlike cattle, in which uterine twists are associated mainly with the delivery period, in mares, in most cases, the problem occurs before parturition [1, 5, 9]. It is assumed that the basic causes leading to the formation of torsion are the movements of the fetus, which tend to adopt the correct presentation before the approaching birth [10]. An additional element conducive to uterine torsion formation is the wallowing of the mare and her rolling over her back. The uterus can twist in either direction. Some studies suggest right-sided torsions are more common in mares [11], while others indicate the frequency of torsions in both directions is similar [8]. However, the direction of the twist is not critical to the issue. Uterine torsion typically ranges from 90° to 360° or more, with 180° torsions being the most common [12]. The severity of pain and the speed of degenerative changes in the uterus primarily depend on the degree of impaired circulation in the organ, so it is directly related to the intensity of the turn. In a study of 19 mares surgically treated for uterine torsion, pathological changes in the uterine wall were observed in 37% of cases with torsions of at least 180°. No abnormalities in the uterine wall or ligaments were noted in cases with torsions below 180° [13]. Twists may occur cranially from the uterine cervix (precervical torsion) or include it (post-cervical torsion). Turns of the first type are more common in mares, distinguishing this species from cattle, in which post-cervical uterine torsion is usually observed [8, 14, 15, 16]. In cases of post-cervical torsions, the problem may manifest as a spiral course of the folds of the vaginal mucosa, even with the partially diagonal arrangement of the vulval fissure and the appearance of asymmetric edema below the vulva covering the thighs, which significantly facilitates the diagnosis of the disease. Precervical uterine torsions do not lead to the described changes, and the only method of diagnosing abnormalities is per rectal examination, during which, by feeling the course of the broad ligaments of the uterus, one can specify the turning direction. In cases where the uterine torsion exceeds 360° or involves the small colon, determining the direction of torsion, even in this examination, may be seriously difficult or even impossible [17, 18].

In diagnostics, it is essential to emphasize the great value of information obtained based on ultrasound examination of the uterus and fetus. The condition of the uterus is determined by conducting a rectal examination, using a linear probe. Attention should be paid to possible swelling and thickening of the uterus wall and CTUP complex, as well as enlargement of the diameter of blood vessels running in the broad ligaments. In turn, the viability of the fetus is most easily checked with a transabdominal probe, examining its heart rate and controlling the transparency of amniotic fluid [10].

In general, two techniques for surgical repositioning of uterine torsion are used: after performing side flank laparotomy (SFL), when the grid incision is made on the side on which the uterus is twisted (in more complicated cases additional second incision could be done on the other side of the horse to facilitate uterine manipulations by second operator) or after laparotomy carried out under general anesthesia through the midline incision (MI). To resolve uterine torsion problems, non-surgical, conservative reposition methods can be carried out by rolling the mare over the back [19] or, if the torsion occurs during parturition and the cervix is partly open, with the Kamerer grip or extremities rotation, but these methods are not discussed in the above paper.

After reviewing the existing literature and consulting with practitioners, the authors determined that operators often favor one treatment technique over another based on personal preferences rather than objective evidence.

This study aims to compare all the pros and cons of both surgical approaches mentioned above, systematize the indications for uterine torsion repositioning, and present the exact protocol that can be useful to qualify a mare with uterine torsion for surgery by the chosen, specific method.

## Materials and methods

The observations concern 21 mares admitted to the hospital, diagnosed with various stages and degrees of uterine torsionsi. As a rule, mares were admitted due to abdominal pain signs, but the final diagnosis of the cause of colic was made in the unit represented by the authors. The accepted mares represented various horse breeds, the most common patient were Arabian mares (No. 12), but the problem also occurred in thoroughbreds (No. 1), half-bred (6), and draft mares (No. 2). In all mares, the uterine torsions were diagnosed in the last trimester of pregnancy, covering the period from 8 months until the date of delivery (in two mares, the problem manifested itself at term and in both cases the reposition of uterus was achieved by rotation of fetus which was possible to be handle through partly open uterine cervix). In one mare, with torsion < 90°, repositioning was done rectally under sedation. These 3 cases were excluded from further analysis and discussion. The first 10 mares were operated on without exception in general anesthesia using MI without considering other surgical techniques for access to the gravid uterus. For MI, mares were sedated by detomidine and butorphanol (each at 0,005 − 0,001 mg/kg IV). General anesthesia was induced with ketamine (2,2 mg/kg IV) and maintained with isoflurane in oxygen and positive pressure ventilation.

As the number of mares admitted with uterine torsion increased, the authors opted to perform surgery on standing mares under local anesthesia in less complex cases (pregnancy duration under 300 days, good general condition, mild colic symptoms, and no suspected abdominal complications). Using a flank approach (grid incision) to access the abdominal cavity, they achieved promising results. In that mare, sedation was carried out with detomidine in bolus (0.01 mg/kg b.w., i.v.), followed by detomidine and butorphanol (20 mg and 15 mg in 1 l of NaCl solution, respectively) in a drip infusion, according to the effect. Local anesthesia was obtained using a mixture of lignocaine 2% and bupivacaine in a 1:1 ratio. 60–70 ml of that solution was administered with a lumbar puncture needle (0.9 mm, length 7 cm), in the incision line subcutaneously and intramuscularly through all abdominal wall layers.

By the time this article was written, of the next 11 mares admitted with uterine torsion, one case with a 90° torsion was corrected rectally under mild sedation. In one mare where torsion occurred at delivery, successful torsion was repositioned per vaginam (both cases mentioned before and not discussed further in the article). SFL operated five mares, and MI operated three mares under general anesthesia. In one additional case, the mare had to be operated on twice from both approaches. This latter case, due to the unusual course, deserves a detailed description: a huge draft mare was admitted to the hospital with colic signs on the 320th day of pregnancy. Despite the suggestion of the hospital team to operate on the mare under general anesthesia by MI, the owner of the mare did not agree to such a procedure. Therefore, an attempt was made to reposition the twisted uterus by SFL, but due to the size of the pregnant uterus and the mare itself, it was not possible to carry it out from this access. Finally, to save the patient, the owner agreed to carry out a second operation by MI. After the mare’s introduction into anesthesia and placement on the operating table in dorsal recumbency to facilitate manipulation of the pregnant uterus, the mare’s rump was partially raised (about 20 cm above the table level), using a crane and hooks (hooked to the straps on the hind limbs) and after the abdominal incision was made, about 30 l of Ringer’s fluid was introduced into the abdominal cavity. In this way, the uterus was repositioned with a still-alive fetus. Unfortunately, the very next day after the procedure, the fetus died. Due to its great size and malposition, it was not possible to carry out labor by manual and force extraction, and there was a need for a cesarean section. The mare survived the treatments without further complications and was discharged after 2 weeks.

Following surgery, all mares received antibiotics and flunixin meglumine (Flunimeg, Scanvet) at 0.4 mg/kg every 12 h for 5 days. Mares that remained pregnant were also administered altrenogest (Regumate, Merck Animal Health) at 0.044 mg/kg orally once daily for 2 weeks post-surgery. In mares where intraoperative circulatory changes in the uterine wall were observed, pentoxifylline p.o. (Trental, Sanofi) was also administered at a dose of 10 mg/kg body weight every 12 h for 7 days after the procedure. In the postoperative period, pregnant mares were examined ultrasonographically every other day to control the condition of amniotic fluid and fetal viability.

### Statistical analysis

Two statistical models were fitted to explore the relationship between explanatory variables and the binary outcomes of survival and delivery. The models aimed to estimate the potential influence of each variable on the outcome, rather than to establish causality, given the limited sample size. In comparison, power analysis indicated that a total sample of 120 mares would be required to detect a medium-sized effect (OR = 1.72) using logistic regression. Notably, both outcomes had a perfect separation since all mares operated using the SFL procedure and survived and delivered the pregnancy. Moreover, only two mares died during MI, meaning the data was imbalanced. These issues pose a serious challenge to statistically investigating the relationships between variables and survival or pregnancy delivery chances. Firth-type logistic regression was employed to address the small dataset and separation observed in some groups, as it is a standard approach for modelling binary outcomes in the presence of such limitations [20].

One mare that underwent both the MI and SFL procedure was removed from the analysis. Given the high correlation between heart rate and packed cell volume (*r* = 0.83), the variables were combined into one binary predictor. Mares with PCV higher than 43 (threshold) or heart rate higher than 52 (median) were classified as being in a worse condition based on the results of these screening tests. Similarly, mares in the 11th month of pregnancy were classified as being in the later stage, considering past evidence showing that surgeries on such mares yield worse results [8, 21].

To reflect the severity of torsion degree, the variable was coded as ordinal with three levels (180, 270, 360). The surgery type was used as a predictor variable in both models. Whether the fetus was dead (or not) was also used as a predictor for survival. In the model for delivery outcome, mares that had a dead fetus were removed from the analysis (*n* = 2). Importantly, no interactions between the surgery type and other variables were included due to further separation issues.

## Results

Chosen clinical data at admission of operated mares and the outcome of treatments are presented in Table 1.

**Table 1:** Table 1

	ADMISSION						OUTCOME			
MI	Pregnancy duration (month)	HR	PCV	CTUP^®^ oedema	Uterine torsion (°/direction L/R)	Dead fetus	Surgery	Mare recovery to the discharge	Post surgery abortion (days)	Full term delivery of a healthy foal
1	8	46	36		180/L			+		+
2	8	56	44		180/L		-	+		+
3	8	48	40		180/R			euthanized	5	
4	8	62	37		270/L			+		+
5	9	45	39		270/R			+		+
6	9	60	41		180/L			+	6	
7	9	52	43		180/R			+		+
8	10	62	46		270/R	+	c.s. due to the dead fetus	+		
9	10	49	39		180/R			+		+
10	10	62	41	+	180/R		c.s. due to the inability of UT reposition	+		
11	11	44	39		180/R			+		+
12	11	86	58	+	360/L	+	c.s. due to the dead fetus	died		
13*	11	64	41		270/R			+	2	
SFL										
1	8	53	40		180/L			+		+
2	8	48	39		180/L			+		+
3	9	42	35		180/L			+		+
4	9	62	43		180/R			+		+
5	9	52	35		180/L			+		+
6	10	45	34		180/L			+		+
7*	11	64	41		270/R		switch to MI due to the inability of UT reposition	+	2	

c.s.- cesarian section

*the same patient operated at first by SFL and then by MI

^®^CTUP noted only when exceed more than 30% of limits for consecutive month of pregnancy (9–10 month < 10 mm; 10–11 < 11 mm; 12 < 12 mm)

Overall success rate: 68,4%

Mares survival rate: 89,5%

Of the seven mares operated on by SFL, six recovered and gave birth to healthy foals. As described earlier, in one of the operated mares, repositioning of the uterus with this technique was unsuccessful, and our team finally resolved the problem after performing a laparotomy in the midline. Out of 13 operated mares by MI (including the last-mentioned case), seven mares fully recovered and gave birth to normal foals. Three other mares aborted a dead fetus during the first week after surgery. Two of these mares, after treatment, were finally released from the hospital in good condition; the third one was euthanized 5 days after the operation because of evisceration through the infected laparotomy wound. Evisceration was a result of severe strainings during an attempt to abort a malpositioned dead fetus. The next mare died during the operation because of shock. This mare, in 10 months of pregnancy, with uterine torsion > 360°, presented at admission with deplorable general condition (HR 78, CRT 3,5 s, PCV 58). Transabdominal ultrasound examination revealed an already dead fetus, CTUP ranging from 14 to 17 mm, and large distended vessels of the broad ligament. The last two mares operated on by MI underwent intraoperative CS as well. One of them was because of a dead fetus at admission, the second because of the inability of uterine reposition due to severe UCTP oedema. Both of these mares recovered well and were released from the hospital.

Neither of the introduced statistical analysis models was significant (*p* = 0.69 and *p* = 0.61), and there was no relationship between a single predictor and the outcomes. This supports the idea that statistical analysis did not produce vastly informative results, likely due to insufficient sample size, rather than a lack of real effects. Overall, undergoing the MI procedure was associated with a non-significant decrease in the odds of survival (OR = 0.31, 95% CI: 0.002, 7.34, *p* = 0.476) and delivery (OR = 0.19, 95% CI: 0.001–3.06, *p* = 0.26). These values are likely inflated due to total separation and the severe clinical condition of some of the mares undergoing MI. The linear trend of the torsion degree was also associated with a non-significant decrease in the odds of survival (*OR* = 0.22, 95% CI: 0.001–31.18, *p* = 0.514). All other log odds in the model fell in the [-0.86, 0.95] range. Overall, no causality can be inferred based on these results.

## Discussion

The use of access from the median incision without exceptions in the first 10 operations was primarily because many horses are operated on in the unit, as represented by the authors, due to gastrointestinal colic. For these reasons, the operators were used to procedures performed under general anesthesia using this approach.

Looking through the literature, it can be noted that individual operators often prefer to use one selected operational technique [13, 22, 23]. This observation and the collection of our own experience [24, 25] allowed for comparing both methods, encouraging the authors to write this publication. First of all, it is worth summarizing and comparing the advantages and disadvantages of each operating method:

Considering repositioning of uterine torsion by SFL, the main advantages of this approach, which should be mentioned, are: short duration, low burdening of the mare and fetus with narcotic drugs, and shorter and safer recovery. The advantage of this method is also the ease of carrying it out in field conditions without specialized facilities, equipment, and additional personnel.

The negative side of SFL is undoubtedly the significant limitation of visual inspection of the abdominal organs. It is also essential that some horses, due to their temperament, may create problems during the procedure. In the authors’ opinion, the size of a mare and the physical possibilities of the operator also should be taken into consideration (repositioning of uterine torsion in a huge mare can turn out to be a problem for short and not physically trained operators, and in these cases, MI will be preferable).

The indisputable advantages of the procedure performed on mares under general anesthesia by MI is undoubtedly a much better possibility of inspecting the abdominal cavity and thus the possibility of assessing the actual condition of the mare’s uterus as well as excluding the coexistence of other problems (most often the part of the digestive system) that may accompany uterine torsion [13]. General anesthesia undeniably provides the operator complete comfort and safety in their work. An additional advantage of performing MI is the ability to manipulate not only the pregnant uterus but also to some extent, the mare on the operating table (twisting the mare to the side and lifting her hind limbs using a crane), which can be helpful in the case of turns that are difficult to reposition. In this recumbency, it will also be much more effective to fill the peritoneal cavity of the operated mare with a large amount of fluid to facilitate the manipulation of the pregnant uterus. Also, in cases where the necessity of performing a cesarean section should be taken into account, according to the authors’ experience, access from the midline is much more advantageous.

The advantage of performing a laparotomy by MI is also the chance of successful surgery just before parturition, and the mare can give birth spontaneously [7].

The undeniable disadvantage of operating from this approach is the need to perform general anesthesia, which is more burdensome for both the mare and the fetus. The vascular compromise resulting from uterine torsion could be further exacerbated by general anesthesia and dorsal recumbency, which may be additional risk factors for the foal when the operation is carried out on the recumbent mare [21]. The MI requires a longer recovery time for the patient before returning to full usability, and what cannot be missed is that the cost of this procedure is much higher.

Other authors’ work, carried out on a large number of procedures and comparing the results of the use of individual techniques to solve uterine torsion in mare, indicates that SFL gives a better chance of success when fetal survival is taken under cionsideration as long as it takes place before the 320th day of gestation [11, 21]. However, even this observation is questionable if we consider the significant advances in anaesthesiology during the period concerning the observations described in the cited papers.

The authors of the paper would also like to refer to the described case of evisceration of a mare under abortion of the foetus, 5 days after the MI procedure. The described case was the only complication of this type for over 1000 horses operated on due to various types of colic (including mares operated on at different stages of pregnancy) in our hospital, so in the authors’ opinion, the risk of this type of complication is extremely low.

Bearing in mind the advantages mentioned above and the disadvantages of both methods and their own experience, currently, the authors use the following type of key, qualifying mares for surgery with one or the other method:

1. Qualification for the procedure on a standing mare by flank laparotomy:
  - A mare of a balanced character, promising approval of the procedure in sedation and local anesthesia.
  - Pregnancy up to 320 days (according to literature and considering that the fetus is growing and gaining weight in a linear pattern almost to the end of pregnancy [26]). The authors believe that this criterion should be treated as an “approximation.” The mare’s absolute size and the fetus’s expected size should be considered in every single case when making the final decision.
  - Diagnosed uterine torsion direction.
  - No apparent advanced circulatory changes within the uterus wall (severe CTUP enlargement) and/or broad ligament (congestive changes in blood vessels) on ultrasound exam.
  - A living fetus without parameters indicating an imminent threat to life.
  - No suspicion of comorbidities (involvement of the digestive system).
2. Qualification for surgery under general anesthesia by midline incision.
  - A mare with reactions difficult to predict, agitated, which, despite sedation, can create problems.
  - Pregnancy over 320 days (mare at term indication if there are no chances for non-surgical intervention).
  - Dead fetus or other situation in which a cesarean section is considered.
  - Suspicion of severely compromised uterine wall and/or broad ligaments of the uterus (severely enlarged CTUP can be a sign that the uterus is prone to breakage during handling, or the necessity to perform hysterectomy may occur).
  - Suspicion of concomitant abdominal problems.

A considerable limitation of the study was the limited data available for the analysis. Because of this, there is no possibility for an objective judgment of the outcome of the SFL vs. IM surgical technique. Future research should address this by systematically comparing the success of each surgical procedure while accounting for other covariates. Moving from exploratory analysis into hypothesis testing would help validate whether MI can yield better results under conditions described in this article.

## Conclusions

In conclusion, it is worth emphasizing that none of the methods is the best, and the key to finding the optimal solution is the appropriate qualification for every case. The choice should be made using objective analysis of clinical data and horse character, whereas surgeons should be prepared every time to proceed with each technique.

## Data Availability

No datasets were generated or analysed during the current study.
